# Meta-analysis and genome-wide interpretation of genetic susceptibility to drug addiction

**DOI:** 10.1186/1471-2164-12-508

**Published:** 2011-10-15

**Authors:** Chuan-Yun Li, Wei-Zhen Zhou, Ping-Wu Zhang, Catherine Johnson, Liping Wei, George R Uhl

**Affiliations:** 1 Laboratory of Bioinformatics and Genomic Medicine, Institute of Molecular Medicine, Peking University, Beijing, China; 2 Center for Bioinformatics, National Laboratory of Protein Engineering and Plant Genetic Engineering, College of Life Sciences, Peking University. Beijing, China; 3 Department of Neurology, the Johns Hopkins School of Medicine, Baltimore, Maryland, USA; 4 Molecular Neurobiology Branch, NIH-IRP (NIDA), Suite 3510, 333 Cassell Drive Baltimore, Maryland, USA

## Abstract

**Background:**

Classical genetic studies provide strong evidence for heritable contributions to susceptibility to developing dependence on addictive substances. Candidate gene and genome-wide association studies (GWAS) have sought genes, chromosomal regions and allelic variants likely to contribute to susceptibility to drug addiction.

**Results:**

Here, we performed a meta-analysis of addiction candidate gene association studies and GWAS to investigate possible functional mechanisms associated with addiction susceptibility. From meta-data retrieved from 212 publications on candidate gene association studies and 5 GWAS reports, we linked a total of 843 haplotypes to addiction susceptibility. We mapped the SNPs in these haplotypes to functional and regulatory elements in the genome and estimated the magnitude of the contributions of different molecular mechanisms to their effects on addiction susceptibility. In addition to SNPs in coding regions, these data suggest that haplotypes in gene regulatory regions may also contribute to addiction susceptibility. When we compared the lists of genes identified by association studies and those identified by molecular biological studies of drug-regulated genes, we observed significantly higher participation in the same gene interaction networks than expected by chance, despite little overlap between the two gene lists.

**Conclusions:**

These results appear to offer new insights into the genetic factors underlying drug addiction.

## Background

Twin and other classical genetic studies indicate that drug addiction is a complex brain disorder with strong genetic contributions [1,2]. Genetic association studies, including candidate gene studies and genome-wide association studies (GWAS), can provide insights into the genetic background of this neurobiological and behavioral disorder. Using these approaches, more than 800 publications during the past three decades have reported genomic loci and/or specific genetic variants that have been associated with susceptibility to drug addiction. It has been difficult to draw general inferences from these studies, however, because genetic association studies generated results that were sometimes inconsistent, many of these studies were modestly powered (especially when polygenic genetic architectures are considered), genomic controls are infrequent, and biases can be detected in a number of analytic strategies. In this context, meta-analysis of genetic association studies may be particularly useful, especially when the underlying genetic architecture for the disorder is relatively straightforward [3-6]. In addition, although different addictive drugs have disparate pharmacological effects, there are also similarities after acute and chronic exposure such as acute rewarding and negative emotional symptoms upon drug withdrawal [7]. It would thus be interesting to elucidate the potential 'common' genetic backgrounds underlying those shared addictive actions, which might further help the development of effective treatments for a wide range of addictive disorders [7,8]. However, to date there have only been limited meta-analyses on drug addiction, mostly focused on candidate genes, and none on GWAS.

Although the number of genetic variations identified has increased rapidly, the understanding of how genetic variations contribute to disease susceptibility has lagged behind. Earlier studies mainly focused on nonsynonymous SNPs [9,10]. More recent studies have attempted to explain functional mechanisms of action of haplotypes that contain SNP and other regulatory variants [11-17]. A number of haplotypes defined by specific SNPs have been found to alter gene expression by modifying transcription factor binding sites [11], microRNA binding sites [12-15] and alternative splicing [16]. Others regulate signaling pathways [17]. However to date there have been only modest genome-scale efforts to study the molecular mechanisms of addiction-associated genetic variants. The relative contributions of different molecular mechanisms remain largely unknown.

Previous work has been spotty in seeking or reporting overlap between the genes identified by genetic association studies and genes identified by other molecular biologic approaches, such as animal models, cDNA microarrays and proteomics [18-20]. Our prior systematic assembly of data obtained by these "other" approaches [21] allows us to seek such overlaps in a systematic fashion.

## Results

### Meta-analysis of genetic association studies of drug addiction

First, we performed an integration and meta-analysis of candidate gene association studies of drug addiction. We retrieved 886 publications on candidate gene association studies of drug addiction from PubMed by keywords query and review paper curation (**See details in Methods**). Two hundred and twelve of these reports met our inclusion criteria, from which we extracted data on 506 allelic contrast tests for 286 genetic variants (Additional file 1). Thirty-five genetic variants were examined in case-control genotype comparisons from three or more independent datasets. We carried out meta-analyses of these 35 genetic variants under simple genetic models using both the random-effects model and fixed-effects model [22,23]. From these data, 12 genetic variants in 11 genes showed effects that reached statistical significance (Table 1, Additional file 2). We noted that most of these variants show comparatively weak genetic effects, with fixed effects summary odds ratios (OR) ranging from 0.52 to 2.34 (Table 1), typical results for studies on other highly heritable phenotypes with "common variants, common disease" design [3-6]. We further assessed the variants using criteria established by the HuGENet Road Map [24] that was recently proposed for assessing cumulative evidence from genetic association studies. Using these stringent criteria, six variants displayed moderate epidemiological credibility (Grade B, Table 1). A full list of the curated information is available online at http://karg.cbi.pku.edu.cn/karg2/. For each study, we extracted meta-data including over thirty demographic and experimental variables where available (Additional file 1).

**Table 1:** Genes and polymorphisms showing significant summary odds ratio (OR) of the addiction susceptibility from random/fixed-effects meta-analyses using allelic contrasts

Gene Name	Polymorphism*	Model (Major allele > Minor Allele)	Cases vs. Controls (independent samples)	Fixed Effects OR (95% CI)	Random Effects OR (95% CI)	Heterogeneity *p-*Value	I- Square	Grade**
SLC4A7	rs3278	G > A	1410 vs. 906 (3)	2.34 (1.599-3.420)	2.28 (1.555-3.333)	0.51	0	B
DRD4	48-bp repeat	Other > 7/8 repeats	2324 vs. 1932 (6)	1.44 (1.155-1.804)	1.48 (1.000-2.197)	0.06	52	C
DRD2/ ANKK1***	Taq1A	A2 > A1	6312 vs. 7424 (20)	1.30 (1.192-1.410)	1.38 (1.096-1.733)	< 0.0001	84	C
BDNF	rs6265	G > A	2530 vs. 4126 (9)	1.31 (1.165-1.451)	1.38 (1.056-1.790)	< 0.0001	80	C
CCK	-45 C/T	C > T	860 vs. 2002 (6)	1.34 (1.089-1.650)	1.34 (1.083-1.646)	0.62	0	B
FAAH	rs324420	P > T	498 vs. 1570 (3)	1.38 (1.014-1.875)	1.32 (0.807-2.171)	0.24	28	B
OPRM1	rs1799971	A > G	2846 vs. 4072 (9)	1.24 (1.090-1.410)	1.31 (0.958-1.790)	< 0.0001	80	C
COMT	rs4680	Val > Met	862 vs. 1594 (3)	0.76 (0.634-0.923)	0.82 (0.644-1.051)	0.71	0	B
CNR1	(AAT)n	14 repeats > Other	2304 vs. 2144 (8)	0.76 (0.658-0.878)	0.75 (0.619-0.906)	0.17	32	B
HNMT	rs35953316	Thr > Ile	1540 vs. 1306 (3)	0.76 (0.598-0.975)	0.72 (0.444-1.179)	0.04	70	C
OPRK1	rs702764	A > G	292 vs. 246 (3)	0.62 (0.431-0.901)	0.62 (0.412-0.944)	0.99	0	B
OPRM1	C691G	C > G	796 vs. 786 (3)	0.52 (0.416-0.647)	0.61 (0.330-1.095)	0.0025	83	C

*Variants were ranked based on the summary ORs. **Degree of 'epidemiological credibility' based on published protocols (A, strong; B, modest; C, weak; see Methods for more details). ***Researchers previously associated the polymorphism Taq 1A to the *DRD2 *gene. However, the polymorphism sits in an exon of the *ANKK1 *gene.

Next, we retrieved 11 independent datasets of drug addiction GWAS [25-31] (**See Details in Methods**). Among them five datasets met our criteria for inclusion [25-27]. We integrated the five GWAS datasets using a new meta-analysis approach to select positive SNPs with significantly more GWAS support than expected by chance (**See Details in Methods**). Overall, 842 SNPs were supported by at least three items of positive evidence, with meta-false discovery rates less than 0.05 (Additional file 3).

We combined the findings identified by candidate gene association studies and GWAS into a list of 849 SNPs in 843 haplotypes. Since many of the genetic susceptibility SNPs may provide genetic 'tag markers', while these tag SNPs were generally designed to detect linkage disequilibrium blocks and functional SNPs may be easily left out in most GWAS platforms [32], we thus used the whole-genome linkage disequilibrium data identified by HapMap [33] to expand the list into 1,907 SNPs by adding SNPs that displayed strong linkage disequilibrium with these genetic marker SNPs in all three HapMap populations.

### Genome-wide analysis of possible molecular mechanisms of the addiction susceptibility factors

We mapped the 1,907 SNPs to putative functional elements in the human genome. As summarized in Table 2 and detailed in Additional file 4, we identified a total of 124 putative functional SNPs in 70 of the haplotype blocks identified herein. Only 26 of these putative functional SNPs, in 23 haplotypes, were non-synonymous. One SNP was located in splicing junctions. Four lay in putative transcription factor binding sites. Two lay in potential microRNA target sites. By integrating data from high-throughput studies that have correlated human genotypes with levels of gene expression (**See Details in Methods**), we found that 24 SNPs in two haplotypes were strongly correlated with differential expression of at least one human gene, one haplotype also contain SNP located in transcription factor binding sites, providing a possible explanation for the observed correlations (Additional file 4).

**Table 2:** Functional categories of addiction susceptibility SNPs

Functional Categories	Vulnerable SNP Number	Haplotype Number	*Monte Carlo* *p*-values
SNPs Introducing Non-Synonymous Mutations	26	23	0.001*
SNPs Introducing Synonymous Mutations	25	21	0.001*
SNPs Introducing Stop Codon Gain	0	0	1.00
SNPs Introducing Stop Codon Lost	0	0	1.00
SNPs Introducing ORF Frame Shift	0	0	1.00
SNPs Introducing Altered Splicing Junction	1	1	0.92
SNPs Introducing Altered TF Binding Sites	4	4	0.83
SNPs Introducing Altered miRNA Targets	2	2	0.28
SNPs Correlated With Differentially Gene Expression	24	2	0.42
SNPs Under Positive Selection	34	19	0.99
SNPs Under Negative Selection	31	26	0.05*
**Functional Addiction Susceptibility SNPs**	124	70	0.63
**All SNPs in The Positive Haplotypes**	1907	843	1.00

Additional evidence for functional roles for many of these SNPs came from studies of apparent effects of natural selection. A total of 31 SNP in 26 haplotypes displayed evidence for negative selection. Thirty-four SNPs in 19 haplotypes displayed evidence for positive selection. Signals of recent positive selection provide information about the adaptation of humans to local conditions and have been implicated in phenotypic variations [34]. Thus, the 6 genes located in these regions of positive selection may be of particular interest in studying addiction vulnerabilities.

We estimated the magnitude of the contributions of different molecular mechanisms to the effects of addiction susceptibility. We compared observed values to those that would be obtained by chance based on 10,000 *Monte Carlo *simulations (**See Methods for details**). The categories of 'synonymous SNP' (*p *= 0.001) and 'non-synonymous SNP' (*p *= 0.001) showed nominally significant over-representation, consistent with the conventional idea that SNPs in coding regions may play important roles in disease susceptibility. In addition, the data suggest regulatory SNPs that modify transcription factor, microRNA binding or alternative splicing sites, may also contribute to addiction susceptibility in addition to those played by non-synonymous SNPs and other allelic variants.

### Genetic association findings and molecular biology findings form significantly more gene interactions

The 124 functional SNPs identified belong to 50 genes. These addiction susceptible genes are enriched in several functional categories such as focal adhesion (hyper-geometric test, *p*-Value = 0.02) that had been previously reported to be involved in drug addiction [21]. We compared these findings to findings from molecular biological studies extracted from the Knowledgebase for Addiction Related Genes (KARG) [21]. In KARG, 348 genes are linked to addiction susceptibility by at least two independent lines of molecular biologic evidence such as results from animal mutagenesis, microarray mRNA profiling and proteomics profiling. Only four genes were common between the two genetic association findings and the molecular biology findings (Official Symbol: FAAH, OPRM1, OPRK1, BDNF), consistent with previously observed modest overlaps between genetic and molecular biology findings in studies of other diseases [35].

We set out to explain this difference with further analysis. Because of the different nature of genetic experiments and molecular biology experiments, could they have discovered different genes in the same molecular network underlying addiction? We hypothesized that the genes identified by genetic studies and those by molecular biology studies may interact more frequently than expected by chance. Indeed, gene interaction enrichment analyses (**See Details in Methods**) revealed that genes identified by these two types of studies interact with each other more than expected by chance. The addiction susceptibility genes formed interactions with 37.2% (89/239) of the addiction-related genes identified by molecular biology studies that had known interaction data (*Monte Carlo p*-value < 0.0001). This result thus provides one explanation for the differences between the genes identified through genetics and those identified through molecular biologic and molecular pharmacologic approaches.

### Development of an updated version of KARG database

We make all of our new data publicly available in an updated version of a comprehensive knowledgebase for addiction-related genes, KARG [21], available at http://karg.cbi.pku.edu.cn/karg2/.

## Discussion

In this study, we collected genetic association studies published in the field of drug addiction for meta-analyses. The power of such meta-analyses is linked to the relatively simple model of the underlying genetic architecture that they presuppose: that SNP genotype results from different samples with differences in genetic background will provide association with drug addiction with the same phase. The significant convergence that such analyses provide herein does support roles for genetic variants with these properties in some aspects of individual differences in susceptibility to dependence. However, recent analyses also provide evidence for roles in addiction susceptibility for more "recent" variants raised after population divergences, which are less likely to be identified by such meta-analytic procedures. Besides the 'common' genetic background identified, it is also interesting to evaluate susceptible variants for different addictive drugs. However, currently the number of available allelic contrast tests data was too limited to perform such an analysis. In the future we will continue to integrate new data toward a better understanding of drug addiction. In addition, recent re-sequencing efforts using next-generation deep sequencing technology support larger effects for at least some rarer variants in both Mendelian [36-40] and complex diseases [41,42], which would also be missed by the current analyses. Nevertheless, the interesting findings from these meta-analyses is complementary to recently published gene-based approach that was used to analyze primary GWAS data in ways that allow for substantial allelic and locus heterogeneities [25-27]. This study also provided an opportunity to study the relationship between addiction susceptible genes identified by traditional genetic association studies and rare addiction causal variants linked by "common disease, rare variants" approaches, when more genomic re-sequencing efforts become available [43-45].

Over 800 candidate gene association studies have been published in this field, but only 212 (24%) of these reports met our inclusion criteria. Some papers published 20 years ago were missing raw genotype and allelic distribution data and had inconsistent use of genetic markers. In addition, since the number of available allelic contrast tests was limited, we combined all data regardless of the types of addictive drugs and the racial/ethnic composition of the group studied. The heterogeneity of the datasets was high: even after our comprehensive meta-analysis, the results were still Grades B and C, according to the criteria of the Human Genome Epidemiology Network (HuGENet) (Table 1). Protocols such as those proposed by HuGENet [24] could standardize data collection and reporting and allow for improved meta-analyses in the future.

Compared to candidate gene association studies, GWAS provide hypothesis-free, genome-wide view of possible genetic susceptibility factors underlying drug addiction [25-31]. When we compare the addiction susceptible genetic variants linked by candidate gene association studies and GWAS, we found that the GWA arrays included probes for three polymorphisms showing significant summary odds ratio of the addiction susceptibility (rs6265, rs1799971 and rs4680). Among these polymorphisms, only rs1799971 show some suggestive significance in methamphetamine abusers of Japanese (p-Value = 0.0465) [26]. Consistent with meta-analyses in Alzheimer disease, schizophrenia, major depressive disorder and Parkinson disease [3-6], it seems some important candidate genes have received inordinate attention in candidate-gene based association study, while the GWA studies with hypothesis-free design might not support many *a prior *hypothesis. On the other hand, GWAS provide more opportunities for traditional candidate-gene based association study to improve the experimental designs by avoiding potential biases from subjectively selection of candidate genes in the beginning of the study.

We were able to tentatively link 124 of the identified susceptibility variations to potential functional mechanisms (Additional file 4). We expanded the genetics tag SNPs using haplotype data to detect the most likely nearly functional SNPs and genes. Besides fitting with the conventional idea that SNPs in coding regions may play important roles in disease susceptibility, the analyses presented here suggest that regulatory SNPs may also play important roles in addiction susceptibility. It will be interesting to study why and how natural selection shaped these *cis*-regulatory factors that potentially modulate addiction susceptibility.

To explain the modest overlap between genetic association findings and other molecular biology findings at the gene level, we identified abundant evidence for interactions between the sets of genes identified in these two ways. Thus, at the level of network analysis, there was good consistency between the genetic and molecular biologic results. This new insight should continue to motivate communication between geneticists and molecular biologists as they study addiction from different perspectives.

## Conclusions

In this study, we report the first comprehensive meta-analysis of genetic association studies in drug addiction. We curated and integrated 212 candidate gene association studies and 5 GWAS. 843 vulnerable haplotypes were identified. We estimated the magnitude of the contributions of different molecular mechanisms to the effects of addiction susceptibility in one of the first 'post-GWAS' global attempts. We further found that at the levels of gene interaction networks, there was in fact good consistency between the genes identified by association studies and those identified by molecular biological studies of drug-regulated genes.

We have made all new data and knowledge publicly available by updating the KARG database [21]. Our study thus provides a 'dynamic' approach. We hope that this approach, as it stands, will provide a basis for meta-analyses of GWAS results of other diseases under the simple genetic architectures postulated herein, as well as a basis for consideration of meta-analytic approaches to more complex architectures in which the focus might be on genes in which variants that display differing frequencies in individuals with different genetic backgrounds are likely to be located. Such analyses could conceivably integrate both the idea of more population-specific variants with the rare variants that are being identified in disease and control samples through re-sequencing efforts.

## Methods

**Figure 1:**
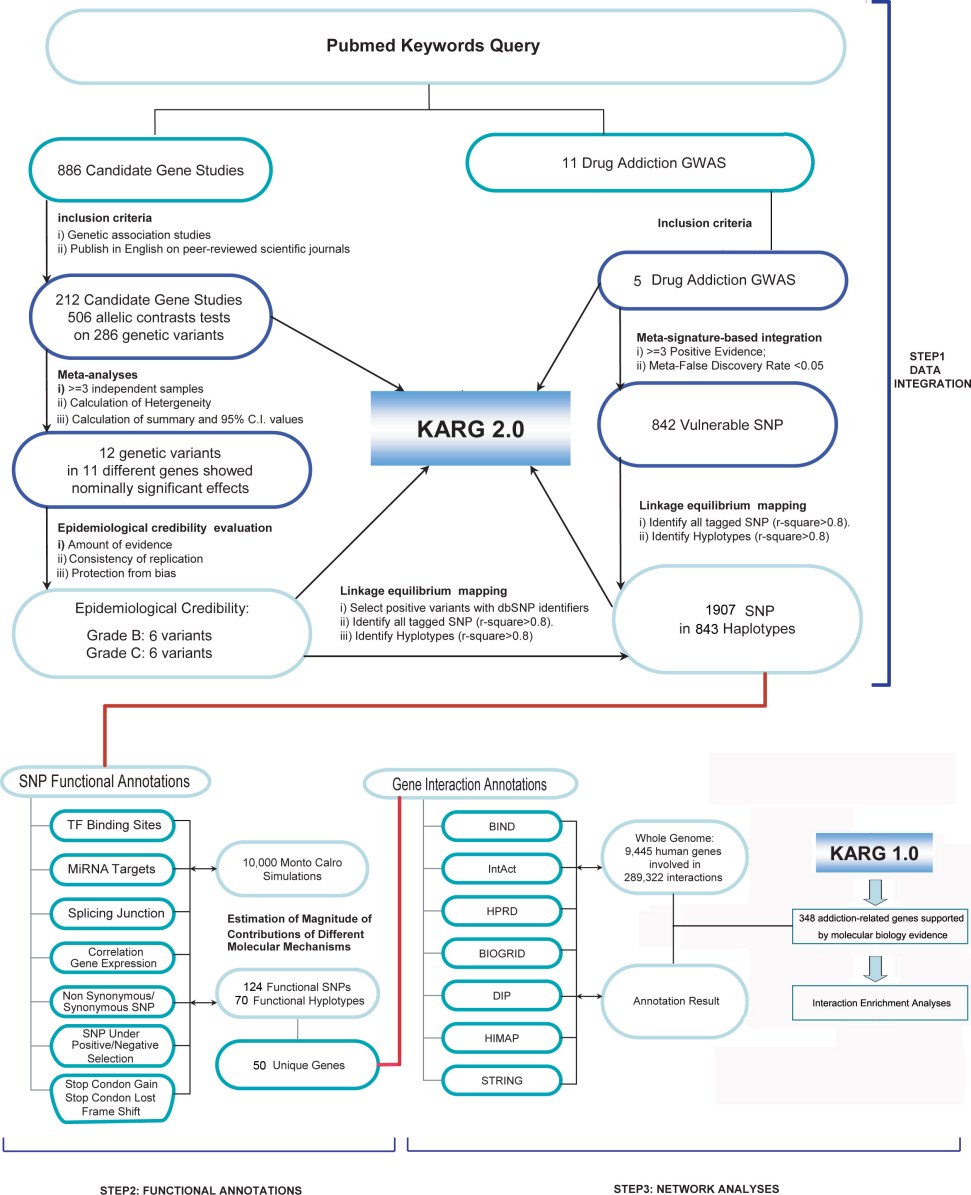
**Pipelines for meta-analyses, functional SNP annotations and interaction analyses**. Meta-analyses of candidate gene association studies and GWAS were illustrated in detail in **STEP 1**. In total, 843 vulnerable haplotypes were identified, linked by 12 risk variants and 842 vulnerable SNPs. All data and knowledge were imported to an updated version of the knowledgebase for addiction-related genes (KARG 2.0, marked with a blue box). Haplotypes identified in **STEP 1 **were annotated with functional and regulatory elements (**STEP 2**). Interaction enrichment analyses between the susceptibility genes and addiction-regulated genes previously identified by molecular biology studies (KARG 1.0, marked with a blue box) were performed (**STEP 3**).

Figure 1 shows the overall pipeline of our meta-analyses of addiction-associated genetic variations, genome-wide analysis of the molecular mechanisms of implicated SNPs, and the pathways and gene interaction networks that might involve these genetic factors.

### Meta-analyses of candidate genetic association studies of drug addiction

To identify the candidate genetic association studies, we performed a search for all abstracts deposited in PubMed database (National Center for Biotechnology Information; NCBI) using the keywords '("addiction" OR "abuse*") AND ("genetic*" AND "association*")'. To identify publications using different phenotype terms other than 'addiction' or 'abuse', we also identify candidate genetic association studies from published reviews selected from PubMed query under the keywords '("addiction" OR "abuse*" OR "dependen*") AND "genetic*" AND Review[ptyp]'. The combined approach resulted in 886 articles. All 886 abstracts were retrieved from PubMed database and manually curated by two independent reviewers. Only those studies that met the following criteria were included in further analyses: (i) It must represent an assessment of association between a polymorphic genetic marker (including SNP and microsatellite markers) and drug addiction phenotypes. Only studies focused exclusively on case-control or population-based designs were included. Studies on markers with more than three alleles (which are generally more difficult to determine unequivocally across different laboratories) or those with an otherwise complex allelic architecture were not considered for meta-analysis. (ii) The study must be published in a peer-reviewed English scientific journal as original research articles. This explicitly excludes studies reported only in the form of an abstract. This yielded 212 papers eligible for inclusion in this study (Figure 1).

From each publication, full text of the original papers were downloaded and manually curated to extracted meta-data, such as publication information ('PubMed ID', 'First Author', 'Title', 'Year' and 'Study Method'), sample information ('Study Design', 'Sample Size,' 'Age', 'Gender Ratio' and 'Ethnic Group'), drug information ('Addictive Drug' and 'Behavior Description'), genotype information ('Gene ID', 'SNP/Marker ID', 'Primary Significance Report', 'Detailed Genotypes in Case and Control' and 'HWE P-value') and curation information ('Curator' and 'Date') (Additional file 1). A full list for the curated information is online available at http://karg.cbi.pku.edu.cn/karg2/.

For all variants with case-control genotype data available in three or more independent samples, we calculated crude ORs (Odds Ratios) and 95% C.I. values of the addiction susceptibility from the allelic distributions for each study following the published protocols [4]. Summary ORs and 95% C.I. values of the addiction susceptibility were then calculated using both the fixed-effects model and DerSimonian & Laird random-effects model [22]. We further graded the epidemiological credibility of these genetic associations according to the criteria of the Human Genome Epidemiology Network (HuGENet) [24]. Details of the grading system followed Ioannidis *et al *[46]. Briefly, each meta-analyzed association is graded on the basis of the amount of evidence, consistency of replication, and protection from bias, following the published protocols [4].

### Meta-analyses of GWAS

On the basis of PubMed query under the keywords 'addiction AND association* AND genome' followed by manually curation, we identified 7 GWAS on drug addiction, containing 11 independent samples. Five of them met our inclusion criteria: i) genetic association studies with case-control design, ii) published in peer-reviewed English scientific journals, iii) the original case-control genotype data is available (raw data available with adequate ethnic approval) and iv) the genotype data are generated by comparable genotyping platforms and arrays with density designs. Detailed raw data of the five GWAS datasets came from the Molecular Neurobiology Branch, NIH-IRP (NIDA) led by Dr. George Uhl, including i) 500 K SNP genotype data from 560 African-American poly-substance abusers who reported dependence on at least one illegal substance and 360 controls [25]; ii) 500 K SNP genotype data from 420 European-American poly-substance abusers who reported dependence on at least one illegal substance and 320 controls [25]; iii) 500 K SNP genotype data from 140 methamphetamine abusers of ethnic Han Chinese origin, with 240 Han Chinese controls [26]; iv) 500 K SNP genotype data from 100 methamphetamine abusers of Japanese origin, with 100 Japanese controls [26] and v) 100 K SNP genotype data from 120 alcohol-dependent individuals and 160 unrelated unaffected controls with European-American ethnicities [27]. Initial data analyses were performed and statistical tests were conducted to assess the susceptibility of each SNP marker [25].

We assumed that results from different GWAS should share a significant intersection of addiction vulnerable SNPs which would be genetic factors underlying drug addiction in general, regardless of addictive drug types and population demographics [32]. We thus implemented a "meta-signature" approach following the "meta-signature" method that Oncomine used to identify common gene-expression signatures [47]. Briefly, (i) Five GWAS as described in the previous paragraph were selected for meta-signature study; (ii) Significant thresholds (T) were chosen to define positive SNPs in the 5 selected GWAS; (iii) Positive SNPs were selected in each GWAS result; (iv) Positive SNPs were sorted by the number of GWAS positive findings in which they are present; (v) the numbers of positive SNPs with 1~5 supporting GWAS were tallied as (T_1_, T_2_, T_3_, T_4_, T_5_); (vi) 10,000 random permutations were performed, in which the actual p-values were randomly assigned to SNPs within each GWAS, so that the positive SNPs in each GWAS change at random, but the number of positive SNPs remained the same. This simulation generated distributions about the number of positive SNPs with 1~5 supporting GWAS, with the means of these distributions tallied as (E_1_, E_2_, E_3_, E_4_, E_5_); (vii) the significance of intersection for the real data was assessed by the minimum meta-false discovery rate (*mFDR*) calculated as *mFDR *= Minimum ([E_*i*_]/[T_*i*_]) for *i *= 1 to *j*, 1 <*j *< = 5. If *mFDR *< 0.05, a meta-signature was defined as those SNPs that are significantly identified (p-value < T) in at least j of 5 independent GWAS, where j is equal to i when mFDR was defined. The p-Value threshold (T) with 0.05 and 0.01 were calculated respectively and significant results were combined for further study. On the basis of the HapMap Linkage Disequilibrium data compiled from genotype data (HapMap data release rel#21 NCBI B35) [33], we further expanded this list using SNP pairs with strong linkage disequilibrium (*r^2^*> = 0.8) in all three HapMap populations. The protocol was implemented in Perl.

### SNP functional annotations

Coordinates of the SNPs were retrieved from NCBI dbSNP database (Build 130) [48]. Genomic coordinates of 3' UTR, 5' UTR, intron region, intergenic regions, synonymous, non-synonymous, and splicing sites were retrieved from the UCSC Genome Browser Database (NCBI36/hg18) [49]. Regulatory elements including transcription factor binding sites and experimentally validated and putative miRNA targets were retrieved from TransFac [50], Argonaute [51], TarBase [14] and PicTar [52]. Information for SNPs under negative selection or positive selection was retrieved from published data [34,49]. The correlation between SNPs and gene expression were retrieved from high-throughput studies correlating human gene expression and genotypes. The full text papers of 11 such studies were manually curated for fulfillment of inclusion criteria of (i) neuropathologically normal samples, (ii) association design and iii) available statistics data. In all, four studies met the inclusion criteria [53-56]. A total list of 33,731 significantly correlated SNP-expression pairs was identified, involving 22,178 SNPs and 3,640 transcripts [53-56]. Then, for the addiction vulnerable SNPs, We estimated the magnitude of the contributions of different molecular mechanisms to the effects of addiction susceptibility. We further compared observed values to those that would be obtained by chance based on 10,000 *Monte Carlo *simulations. Briefly, the positive SNPs were randomly selected from all tag SNPs, but the number of positive SNPs remained the same. Then, for each SNP list, we performed the identical pipelines to estimate the contributions of different molecular mechanisms to the effects of addiction susceptibility. Perl and R scripts were implemented to integrate the datasets, annotate addiction vulnerable SNPs and perform statistical tests.

### Functional enrichment analyses

Information about gene interactions comes from seven interaction databases including IntAct [57], BIND [58], HPRD [59], BioGRID [8], HiMAP, DIP and STRING [60]. We annotated all addiction susceptibility genes using these data. 10,000 *Monte Carlo *simulations were performed to estimate the distribution for testing the enrichment for interactions between addiction susceptibility genes and addiction-related genes identified by molecular biology studies, in which addiction susceptibility gene lists were randomly created from human genome, followed by the identical analyses pipelines for gene interaction annotations and calculations. *Monte Carlo p*-values < 0.05 were considered to be a sign for interaction enrichment between the two datasets to a statistically significant degree. We performed functional enrichment test for addiction susceptibility genes using KOBAS [61] and DAVID [62], following published protocols [21]. Functional categories with *p*-values < 0.05 were considered enriched in addiction susceptibility genes to a statistically significant degree.

### Development of an updated version of KARG database

We updated KARG with the new data and knowledge discussed above. Cross-references to key external databases were included to integrate functional information about the genes, such as gene annotations [49], Gene Ontology (GO) annotations [63], interacting proteins [8,58,59] and functional domain annotations [64]. We enhanced the web-based user interface of the database using PHP and queries of the database using PHP/SQL query script.

## Authors' contributions

CYL, LW and GU conceived and designed the experiments. CYL performed most of the experiments. CYL, WZZ, PWZ and CJ analyzed the data and performed the statistical analysis. CYL, LW and GU wrote the paper. All authors read and approved the final manuscript.

## Supplementary Material

Additional file 1 **Description of meta-data**. Features integrated for each item of evidence. Click here for file

Additional file 2 **Forest plots of meta-analyses**. Forest plots of meta-analyses using allelic contrasts for variations showing significant summary Odds Ratios (OR). Click here for file

Additional file 3 **Vulnerable SNPs identified by Meta-analyses of public GWAS**. Meta-analyses of five genome-wide association studies (GWAS) identified 842 vulnerable SNPs for drug addiction. Click here for file

Additional file 4 **Functional annotations of addiction susceptibility SNPs**. Addiction susceptibility variants and items of evidence. Click here for file
