# Supplementary material for: Meta-analysis and genome-wide interpretation of genetic susceptibility to drug addiction
Source: BMC Genomics. 2011 Oct 15;12:508. doi: 10.1186/1471-2164-12-508 (PMC3215751; doi:10.1186/1471-2164-12-508)
Supplement: Additional file 1 — Description of meta-data. Features integrated for each item of evidence. [file 1471-2164-12-508-S1.DOC]

Additional File 1. Features integrated for each item of evidence

| **Functional Categories** | **Integrated Features** |
| --- | --- |
| Paper Information | PMID; First Author; Title; Year; Method |
| **Sample Information** | Technology Platforms, Study Design (Family-based or Case-control); Case Age; Case Gender Ratio (M%); Control Age; Control Gender Ratio (M%); Ethnic Group (African origin/Asian/Caucasian/Hispanic/Other or Mixed); Case Sample Count; Control Sample Count |
| **Drug Information** | Addictive Drugs; Behavior; Diagnosis Protocols |
| **Genotype Allele Information** | Entrez Gene ID; SNP/Marker ID; Variation Description; Original Reports (Positive/Negative); Minor Allele Frequency in Case; Major Allele Frequency in Case; Minor Allele Frequency in Control; Major Allele Frequency in Control; Control HWE Test P-value |
| **Detailed Genotype Information** | AA in case; AB in Case; BB in Case; AA in Control; AB in Control; BB in Control (A: minor allele, B: major allele) |
| **Curation Information** | Curator; Curation Date |
