# Supplementary material for: Meta-analysis and genome-wide interpretation of genetic susceptibility to drug addiction
Source: BMC Genomics. 2011 Oct 15;12:508. doi: 10.1186/1471-2164-12-508 (PMC3215751; doi:10.1186/1471-2164-12-508)
Supplement: Additional file 2 — Forest plots of meta-analyses. Forest plots of meta-analyses using allelic contrasts for variations showing significant summary Odds Ratios (OR). [file 1471-2164-12-508-S2.DOC]

**Additional File 2**. Forest plots of meta-analyses using allelic contrasts for variations showing significant summary Odds Ratios (OR). For each individual association study, odds ratios and 95% CI were calculated and highlighted with black diamonds with blue bars. The sizes of diamonds were proportional with the total sample sizes. Summary OR with 95% CI was then calculated and highlighted at the end of each forest plot.

**Additional File 2.1** Meta-analysis of ***SLC4A7* rs3238 (G/A)**

0.01

100.0

1

Ishiguro H

2.05 (1.28 - 3.29)

Ishiguro H

4.42 (1.33 - 14.66)

Ishiguro H

2.28 (1.06 - 4.90)

**OR (95% CI)**

Random Effects Model

Summary OR (95% CI) = 2.28 (1.55 to 3.33)

**Odds Ratio (95% CI)**

**Additional File 2.2** Meta-analysis of ***DRD4* 48-bp repeat**

0.01

100.0

1

Adamson MD

0.88 (0.54 - 1.44)

Li T

11.36 (0.65 - 199.28)

Kotler M

2.55 (1.40 - 4.63)

Franke P

1.41 (1.01 - 1.98)

Li T

6.41 (0.31 - 134.22)

Ballon N

1.29 (0.75 - 2.20)

**OR (95% CI)**

Random Effects Model

Summary OR (95% CI) = 1.48 (1.00 to 2.20)

**Odds Ratio (95% CI)**

**Additional File 2.3** Meta-analysis of ***DRD2* Taq1A (A2/A1)**

0.01

100.0

1

Ballon N

1.23 (0.78 - 1.93)

Radwan GN

0.57 (0.39 - 0.85)

Goldman D

3.98 (2.81 - 5.64)

Goldman D

1.16 (0.88 - 1.53)

Geijer T

0.92 (0.50 - 1.68)

O'Hara BF

1.58 (1.09 - 2.28)

O'Hara BF

0.85 (0.57 - 1.28)

Smith SS

1.63 (0.78 - 3.39)

Anghelescu I

1.09 (0.72 - 1.65)

Goldman D

0.71 (0.23 - 2.22)

Comings DE

1.04 (0.75 - 1.45)

Comings DE

1.79 (1.33 - 2.41)

Blum K

9.21 (3.29 - 25.79)

Shahmoradgoli Najafabadi M

4.11 (2.45 - 6.91)

Chen WJ

1.12 (0.85 - 1.47)

Gorwood P

0.92 (0.57 - 1.50)

Lawford BR

4.91 (1.46 - 16.52)

Pastorelli R

1.23 (0.61 - 2.49)

Xu K

0.97 (0.72 - 1.31)

Xu K

1.10 (0.89 - 1.35)

**OR (95% CI)**

Random Effects Model

Summary OR (95% CI) = 1.38 (1.10 to 1.73)

**Odds Ratio (95% CI)**

**Additional File 2.4** Meta-analysis of ***BDNF* rs6265 (G/A)**

**Odds Ratio (95% CI)**

0.01

100.0

1

Matsushita S_a

0.81 (0.66 - 1.00)

Matsushita S_b

1.68 (1.13 - 2.50)

Matsushita S_c

1.52 (1.12 - 2.07)

Matsushita S_d

1.64 (1.05 - 2.57)

Liu QR

0.57 (0.30 - 1.10)

Itoh K

1.96 (1.49 - 2.58)

Lang UE

1.91 (1.21 - 3.03)

Cheng CY_a

1.56 (1.07 - 2.27)

Cheng CY_b

1.30 (0.94 - 1.79)

Random Effects Model

Summary OR (95% CI) = 1.37 (1.06 to 1.79)

**Additional File 2.5** Meta-analysis of ***CCK* -45 polymorphism (C/T)**

0.01

100.0

1

Okubo T

1.36 (0.85 - 2.16)

Okubo T

0.99 (0.57 - 1.71)

Okubo T

1.44 (0.73 - 2.87)

Okubo T

1.23 (0.84 - 1.81)

Comings DE

1.98 (1.16 - 3.39)

Vanakoski J

1.29 (0.68 - 2.47)

**OR (95% CI)**

Random Effects Model

Summary OR (95% CI) = 1.33 (1.08 to 1.65)

**Odds Ratio (95% CI)**

**Additional File 2.6** Meta-analysis of ***FAAH* rs324420 (P/T)**

0.01

100.0

1

Flanagan JM

0.82 (0.38 - 1.76)

Flanagan JM

3.40 (0.46 - 24.91)

Flanagan JM

1.51 (1.06 - 2.14)

Random Effects Model

Summary OR (95% CI) = 1.32 (0.81 to 2.17)

**OR (95% CI)**

**Odds Ratio (95% CI)**

**Additional File 2.7** Meta-analysis of ***OPRM1* rs1799971 (A/G)**

0.01

100.0

1

Franke P

1.11 (0.80 - 1.55)

Franke P

0.97 (0.68 - 1.40)

Bart G

2.31 (1.37 - 3.88)

Bergen AW

1.25 (0.78 - 2.01)

Bergen AW

1.94 (1.29 - 2.93)

Ide S

0.76 (0.49 - 1.18)

Ide S

0.83 (0.60 - 1.13)

Kapur S

3.33 (2.16 - 5.15)

Zhang D

1.01 (0.68 - 1.50)

Random Effects Model

Summary OR (95% CI) = 1.31 (0.96 to 1.79)

**OR (95% CI)**

**Odds Ratio (95% CI)**

**Additional File 2.8** Meta-analysis of ***COMT* rs4680 (Val/Met)**

**Odds Ratio (95% CI)**

0.01

100.0

1

Li T

0.84 (0.63 - 1.14)

Li T

0.72 (0.56 - 0.93)

Nikoshkov A

1.52 (0.65 - 3.54)

Random Effects Model

Summary OR (95% CI) = 0.82 (0.64 to 1.05)

**OR (95% CI)**

**Additional File 2.9** Meta-analysis of ***CNR1* (AAT)n**

0.01

100.0

1

Zhang PW

0.59 (0.45 - 0.77)

Zhang PW

0.48 (0.27 - 0.84)

Comings DE

0.90 (0.54 - 1.48)

Comings DE

0.76 (0.44 - 1.31)

Comings DE

1.00 (0.34 - 2.93)

Ballon N

0.82 (0.53 - 1.27)

Ballon N

0.69 (0.39 - 1.20)

Li T

0.97 (0.75 - 1.25)

Random Effects Model

Summary OR (95% CI) = 0.75 (0.62 to 0.91)

**OR (95% CI)**

**Odds Ratio (95% CI)**

**Additional File 2.10** Meta-analysis of ***HNMT* rs35953316 (Thr/Ile)**

0.01

100.0

1

Reuter M

1.12 (0.75 - 1.66)

Reuter M

0.64 (0.45 - 0.92)

Reuter M

0.45 (0.22 - 0.91)

Random Effects Model

Summary OR (95% CI) = 0.72 (0.44 to 1.18)

**OR (95% CI)**

**Odds Ratio (95% CI)**

**Additional File 2.11** Meta-analysis of ***OPRK1* rs702764 (A/G)**

0.01

100.0

1

Yuferov V

0.61 (0.30 - 1.22)

Yuferov V

0.63 (0.33 - 1.23)

Yuferov V

0.63 (0.28 - 1.42)

Random Effects Model

Summary OR (95% CI) = 0.62 (0.41 to 0.94)

**OR (95% CI)**

**Odds Ratio (95% CI)**

**Additional File 2.12** Meta-analysis of ***OPRM1* C691G**

0.01

100.0

1

Bergen AW

1.30 (0.64 - 2.63)

Bergen AW

0.35 (0.24 - 0.50)

Bergen AW

0.59 (0.44 - 0.81)

Random Effects Model

Summary OR (95% CI) = 0.60 (0.33 to 1.09)

**OR (95% CI)**

**Odds Ratio (95% CI)**
