# Supplementary material for: Meta-analysis and genome-wide interpretation of genetic susceptibility to drug addiction
Source: BMC Genomics. 2011 Oct 15;12:508. doi: 10.1186/1471-2164-12-508 (PMC3215751; doi:10.1186/1471-2164-12-508)
Supplement: Additional file 3 — Vulnerable SNPs identified by Meta-analyses of public GWAS. Meta-analyses of five genome-wide association studies (GWAS) identified 842 vulnerable SNPs for drug addiction. [file 1471-2164-12-508-S3.DOC]

**Additional File 3. Meta-analyses of five genome-wide association studies (GWAS) identified 842 vulnerable SNPs for drug addiction**

| ***p-Value* Cutoff** | **Independent Study** | **Significant SNP** | **Expected SNP (mean)** | **mFDR** |
| --- | --- | --- | --- | --- |
| **0.05** | 1 | 73704 | 200065 | N.S^$^. |
|  | 2 | 74733 | 19980 | N.S. |
|  | 3 | 6182 | 880 | N.S. |
|  | **4** | **249** | **12** | **0.048^*^** |
| **0.01** | 1 | 18385 | 68140 | N.S. |
|  | 2 | 25805 | 1826 | N.S. |
|  | **3** | **618** | **19** | **0.031^*^** |

**$ N.S.=Not Significant; *mFDR<=0.05;**
